# Multifunctional Silica-Based Amphiphilic Block Copolymer Hybrid for Cu(II) and Sodium Oleate Adsorption in Beneficiation Wastewater

**DOI:** 10.3390/polym14194187

**Published:** 2022-10-06

**Authors:** Jia Qu, Liangliang Chang, Mingbao Liu, Baoyue Cao, Meilan Li, Qiang Yang, Wei Gong

**Affiliations:** Shaanxi Key Laboratory of Comprehensive Utilization of Tailings Resources, Shaanxi Engineering Research Center for Mineral Resources Clean & Efficient Conversion and New Materials, Shangluo University, Shangluo 726000, China

**Keywords:** silica, amphipathic, block copolymer, adsorption, beneficiation wastewater, Cu(II), sodium oleate

## Abstract

Beneficiation wastewater contains various types of pollutants, such as heavy metal ions and organic pollutants. In this work, a silica-based amphiphilic block copolymer, SiO_2_–g–PBMA–b–PDMAEMA, was obtained by surface-initiated atom transfer radical polymerization (SI-ATRP) for Cu(II) and sodium oleate adsorption in beneficiation wastewater, using butyl methacrylate (BMA) as a hydrophobic monomer and 2-(dimethylamino)ethylmethacrylate (DMAEMA) as a hydrophilic monomer. FTIR, TGA, NMR, GPC, XRD, N_2_ adsorption–desorption isotherms and TEM were used to characterize the structure and morphology of the hybrid adsorbent. The introduction of PBMA greatly increased the adsorption of sodium oleate on SiO_2_–g–PBMA–b–PDMAEMA. Adsorption kinetics showed that the adsorption of Cu(II) or sodium oleate on SiO_2_–g–PBMA–b–PDMAEMA fitted the pseudo-second-order model well. Adsorption isotherms of Cu(II) on SiO_2_–g–PBMA–b–PDMAEMA were better described by the Langmuir adsorption isotherm model, and sodium oleate on SiO_2_–g–PBMA–b–PDMAEMA was better described by the Freundlich adsorption isotherm model. The maximum adsorption capacity of Cu(II) and sodium oleate calculated from Langmuir adsorption isotherm equation reached 448.43 mg·g^−1^ and 129.03 mg·g^−1^, respectively. Chelation and complexation were considered as the main driving forces of Cu(II) adsorption, and the van der Waals force as well as weak hydrogen bonds were considered the main driving forces of sodium oleate adsorption. The adsorbent was recyclable and showed excellent multicomponent adsorption for Cu(II) and sodium oleate in the mixed solution. SiO_2_–g–PBMA–b–PDMAEMA represents a satisfying adsorption material for the removal of heavy metal ions and organic pollutants in beneficiation wastewater.

## 1. Introduction

Mineral resources are essential and precious sources of wealth for the development of human society. However, in the process of exploitation of mineral resources, a large amount of industrial wastewater will inevitably be produced. If the wastewater is not properly discharged, it will have a serious impact on plant growth, animal fitness and human health, and can also cause serious damage to the local environment and ecology [1,2]. Thus, it is very important to properly treat the wastewater produced during mineral processing.

Among various water treatment technologies, adsorption is widely used because of its operability and high efficiency [3,4]. The adsorption of heavy metal ions from mineral-processing wastewater has been widely studied [5,6,7]. In addition to harmful heavy metal ions, mineral-processing wastewater also contains organic pollutants such as kerosene [8], xanthate [9], aerofloat [10], sodium oleate [11], etc. However, limited research focuses on the simultaneous removal of heavy metal ions as well as organic pollutants from beneficiation wastewater. Therefore, it is necessary to develop a suitable adsorbent material and evaluate its adsorption capacity for both heavy metal ions and organic pollutants.

Copper is widely used in electrical engineering, light industry, machinery manufacturing, construction, national defense and many other fields for its ductility, thermal conductivity and electroconductibility [12,13]. Despite being an essential micronutrient, copper can cause human poisoning at high concentrations, leading to illness and even death [14]. Sodium oleate is a commonly used collector in the mineral processing of oxidized copper ore [15,16]. Sodium oleate will increase the biochemical oxygen demand of water, which may lead to the extinction of aquatic organisms, and will also affect the soil and plant growth around the water [17]. The adsorption removal of copper has been reported in many works in the literature, and the adsorbents involved include activated carbon [18], carbon nanocomposites [19], mesoporous silica [20], zeolite [21], polymer [22], organic–inorganic hybrids [23], etc. However, very few works in the literature deal with the adsorption of sodium oleate. The only adsorbents we have seen in the literature are modified Ca-montmorillonite [24] and Zr-modified phosphogypsum/fly ash composite [25]. Therefore, it is of practical significance to study the removal of copper ions as well as sodium oleate.

Organic–inorganic hybrids are widely used in wastewater treatment for their durability, stability and variable adsorption driving forces [26,27,28]. Gao et al., reported silica-based functional composite particles grafting with poly(2-(dimethylamino)ethyl methacrylate) (PDMAEMA) by solution polymerization, and the hybrid was used for Cr(VI) and Cu(II) adsorption [29]. Zhou et al., reported PDMAEMA brushes on silica particles synthesized by surface-initiated atom transfer radical polymerization (SI-ATRP), and the hybrid was used for Cr(VI) adsorption [30]. Wang et al., reported a kapok fiber coated by a mixture of polybutylmethacrylate (PBMA) and hydrophobic silica, and the coated fiber was used for oil adsorption [31]. In this work, a new kind of silica-based amphiphilic block copolymer hybrid adsorbent, SiO_2_–g–PBMA–b–PDMAEMA, was prepared by SI-ATRP and was used for Cu(II) and sodium oleate adsorption. DMAEMA and BMA were used as the hydrophilic and hydrophobic monomer, respectively. FTIR, TGA, NMR, GPC, XRD, N_2_ adsorption–desorption isotherms and TEM were used to characterize the structure and morphology of the hybrid adsorbent. The adsorption kinetics and adsorption isotherms of Cu(II) and sodium oleate in water were studied, the simultaneous adsorption was taken into account, and the possible adsorption mechanism was proposed. This study is of practical significance to the removal of heavy metal ions and organic pollutants from mineral-processing wastewater.

## 2. Materials and Methods

### 2.1. Materials

The materials and the corresponding purification methods are shown in Table 1.

### 2.2. Preparation of Silica Initiator SiO_2_–Br

The preparation of the initiator was carried out in two steps [33].

**Scheme 1:**
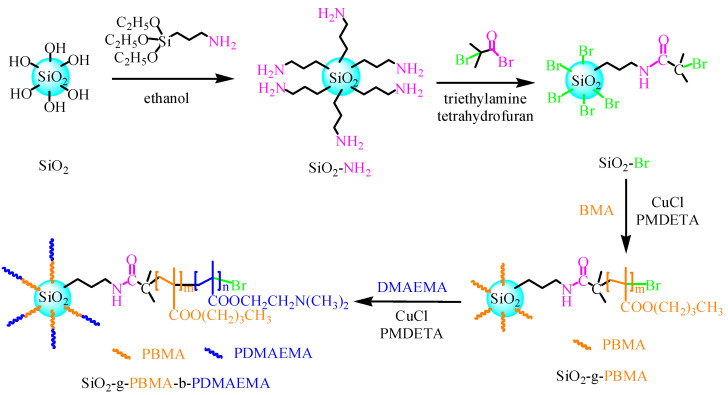
Synthesis of SiO_2_–g–PBMA–b–PDMAEMA.

The first step was the amination of SiO_2_. SiO_2_ (2.7 g) and co-solvent (87 mL deionized water and 63 mL ethanol) were added to a three-necked flask and dispersed by sonication combined with mechanical agitation. The ethanol solution of APTES (10.5 mL APTES dissolved in 24 mL ethanol) was then dropped into the three-necked flask and the pH of reaction mixture was set to 10 with ammonia. The reaction was conducted at 50 ℃ for 24 h to obtain the crude product. The amino-modified nano-silica (SiO_2_–NH_2_) was obtained after washing with deionized water and ethanol alternately and vacuum drying at 50 ℃. The second step was to graft bromine atoms on the surface of SiO_2_–NH_2_ to obtain the initiator. SiO_2_–NH_2_ (0.5 g) was dispersed in THF (10 mL) in a dried Schlenk flask by ultrasound. Under an ice bath, the Schlenk flask was filled with N_2_ and the suspension was stirred for 30 min. Triethylamine (1.5 mL) was injected, and then the mixture of 2-bromoisobutyryl bromide (3 mL) and THF (40 mL) was added dropwise. The reaction was kept in an ice bath for 4 h, and then kept at 35 ℃ for 48 h. The silica initiator (SiO_2_–Br) was obtained after washing with deionized water and ethanol alternately and vacuum drying at 50 ℃. The synthesis scheme of the silica initiator SiO_2_–Br is given in Scheme 1.

### 2.3. Preparation of SiO_2_–g–PBMA–b–PDMAEMA Hybrid by SI-ATRP

One-step ATRP was used to obtain the SiO_2_–g–PBMA–b–PDMAEMA hybrid. CuCl and CuCl_2_ were added into a dried Schlenk flask, and the flask was filled with N_2_ before the sequential injection of BMA, PMDETA and solvent with initiator (SiO_2_–Br dispersed in cyclohexanone). It took 24 h for the grafting of BMA onto SiO_2_ at 90 ℃. The cyclohexanone solution of DMAEMA was injected into the flask after cooling the reaction system to 70 ℃, and it took 24 h for the grafting of DMAEMA onto SiO_2_–g–PBMA at 70 ℃. SiO_2_–g–PBMA–b–PDMAEMA was obtained after purification with THF and vacuum drying at 50 ℃. The synthesis scheme of SiO_2_–g–PBMA–b–PDMAEMA is also given in Scheme 1, and the detailed recipes of polymerization are listed in Table 2.

### 2.4. Characterization of Initiator and Hybrids

Fourier transform infrared (FTIR) spectra were collected by FTIR spectroscopy (Nicolet-380, Thermo Electron Corporation, Waltham, MA, USA) in a spectral range of 4000–400 cm^−1^. Thermogravimetric curves were obtained by a thermogravimetric analyzer (TGA, STA449, NETZSCH, Selb, Germany) under a N_2_ atmosphere using a heating rate of 10 ℃·min^−1^ from 25 ℃ to 1000 ℃. The nuclear magnetic resonance (^1^H NMR) spectrum of SiO_2_–g–PBMA–b–PDMAEMA (etching with hydrofluoric acid to obtain PBMA–b–PDMAEMA) was collected by NMR spectroscopy (Bruker Avance NEO 600, Bruker, Karlsruhe, Germany) using deuterium generation of chloroform (CDCl_3_) as solvent. The molecular weight of SiO_2_–g–PBMA–b–PDMAEMA (etching with hydrofluoric acid to obtain PBMA–b–PDMAEMA) was measured by a gel permeation chromatograph (GPC, Waters 1525, Waters, Milford, CT, USA) using THF as the eluent with a flow rate of 0.5 mL·min^−1^. X-ray diffraction (XRD) patterns were recorded on an X-ray diffractometer (X’Pert Powder, Panalytical, Almelo, The Netherlands) with Ni-filtered Cu Kα radiation (40 kV, 40 mA) in a 2 theta of 20–80°. Nitrogen adsorption–desorption isotherms were measured at 77 K with a specific surface area and porosity analyzer (ASAP 2460, Micromeritcs, Norcross, GA, USA). The surface area was determined based on the Brunauer–Emmett–Teller (BET) method. Mesoporous size distribution was derived from the desorption branches of the isotherms based on the Barrett–Joyner–Halenda (BJH) model. Transmission electron microscopy (TEM, Talos F200X, FEI, Hillsboro, OR, USA) images were recorded after ultrasounding in water for 30 min at an acceleration voltage of 200 kV.

### 2.5. Adsorption Kinetics

Adsorption kinetics experiments were conducted in batch mode with a shaking speed of 120 rpm under a pH of 5, temperature of 25 ℃, adsorbent dosage of 1 g·L^−1^, initial Cu(II) or sodium oleate concentration of 100 mg·L^−1^ and controlled time *t* (5, 10, 20, 30, 60, 90, 120, 180 and 240 min). After centrifugation, the concentration of Cu(II) residue was determined using an atomic absorption spectrometer (AAS, AA-7003, EWAI, Beijing, China). The concentration of sodium oleate residue was determined using an ultraviolet–visible spectrophotometer (UV–Vis, Cary 5000, Agilent, Palo Alto, CA, USA) with maximum absorption wavelength of 225 nm (Supporting Information, Figure S1) [24,25].

The equilibrium absorption capacity *Q*_e_ (mg·g^−1^) was calculated according to Equation (1) [34]. The removal efficiency *η* (%) was calculated according to Equation (2) [35]:

*Q* = (*C*_0_ − *C*_t_)*V*/*W* (1)

*η* = 100(*C*_0_ − *C*_t_)/*C*_0_ (2)

Here, *C*_0_ (mg·L^−1^) and *C*_t_ (mg·L^−1^) refer to the initial concentrations and equilibrium concentrations of Cu(II) or sodium oleate, respectively, *V* (L) refers to the volume of the solution and *W* (g) refers to the weight of the adsorbent.

Adsorption rate was analyzed using the pseudo-first-order model expressed by Equation (3) [34] and pseudo-second-order model expressed by Equation (4) [34] as follows:

ln(*Q*_e_ − *Q*_t_) = −*k*_1_*t* + ln*Q*_e_ (3)

*t*/*Q*_t_ = *t*/*Q*_e_ + 1/(*k*_2_*Q*_e_^2^) (4)

Here, *Q*_e_ (mg·g^−1^) and *Q*_t_ (mg·g^−1^) are the equilibrium absorption capacity and absorption capacity at time *t* (min), respectively, *k*_1_ (min^−1^) is the pseudo-first-order rate constant and *k*_2_ (g·mg^−1^·min^−1^) is the pseudo-second-order rate constant.

### 2.6. Adsorption Isotherms

Adsorption isotherm experiments were conducted in batch mode with a shaking speed of 120 rpm under a pH of 5, temperature of 25 ℃, adsorbent dosage of 1 g·L^−1^, equilibrium adsorption time obtained from adsorption kinetics, and controlled initial concentration of Cu(II) or sodium oleate. For Cu(II), 20, 50, 100, 500 and 1000 mg·L^−1^ were set to be the initial concentration. For sodium oleate, 20, 40, 60, 80 and 100 mg·L^−1^ were set to be the initial concentration. After equilibrium, the supernatant by centrifugation was analyzed for the concentration of residual Cu(II) or sodium oleate.

The Langmuir isotherm model and the Freundlich isotherm model were investigated. The Langmuir model is expressed by Equation (5) [34]:

*C*_e_/*Q*_e_ = 1/(*Q*_m_*K*_L_) + *C*_e_/*Q*_m_ (5)

Here, *Q*_e_ is the equilibrium adsorption capacity (mg·g^−1^), *C*_e_ is the equilibrium concentration in the solution (mg·L^−1^), *Q*_m_ is the maximum adsorption capacity (mg·g^−1^) and *K*_L_ is the Langmuir adsorption isotherm constant (L·mg^−1^).

The Freundlich model is expressed by Equation (6) [34]:

ln*Q*_e_ = ln*K*_f_ + (1/*n*)ln*C*_e_ (6)

Here, *Q*_e_ is the equilibrium adsorption capacity (mg·g^−1^) and *C*_e_ is the equilibrium concentration in the solution (mg·L^−1^). *K*_f_ is the Freundlich adsorption isotherm constant ((mg·g^−1^) (L·mg^−1^)^1/n^). The term 1/*n* is an empirical constant related to the adsorption driving force.

### 2.7. Recovery Experiments

Three adsorption–desorption cycles were performed. The adsorption of Cu(II) or sodium oleate was carried out at a pH of 5, temperature of 25 ℃, adsorbent dosage of 1 g·L^−1^ and initial Cu(II) or sodium oleate concentration of 100 mg·L^−1^. An amount of 0.1 mol·L^−1^ nitric acid and ethanol were used for the desorption of Cu(II) and sodium oleate, respectively. The adsorbent was washed with deionized water and vacuum-dried before use.

### 2.8. Multicomponent Adsorption

In order to investigate the practicability of the adsorbent, the simultaneous adsorption of Cu(II) and sodium oleate was also taken into account. Multicomponent adsorption experiments were carried out in batch mode with a shaking speed of 120 rpm under a pH of 5, temperature of 25 ℃ and adsorbent dosage of 1 g·L^−1^. A mixture of Cu(II) and sodium oleate at equal concentrations was investigated. The adsorption was carried out for 4 h to ensure adsorption equilibrium. The concentration of Cu(II) residue was determined using an atomic absorption spectrometer, and the concentration of sodium oleate residue was determined using a UV–Vis spectrophotometer with a maximum absorption wavelength of 225 nm. The equilibrium absorption capacity *Q*_e_ (mg·g^−1^) was calculated according to Equation (1), and the removal efficiency *η* (%) was calculated according to Equation (2).

## 3. Results and Discussion

### 3.1. Adsorbent Characterizations

**Figure 1:**
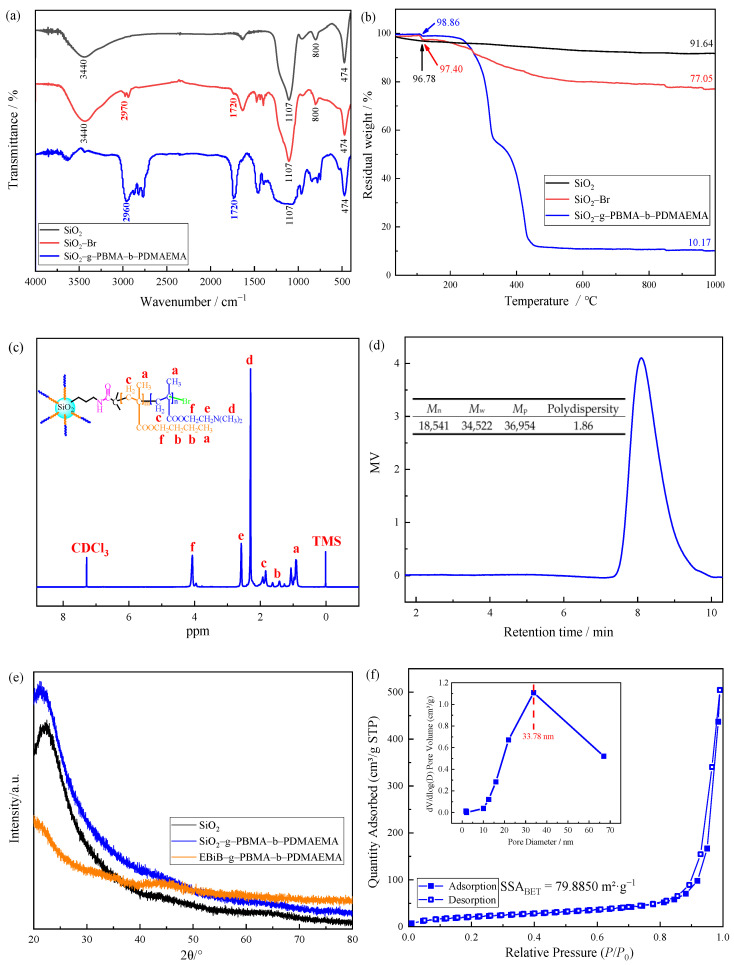

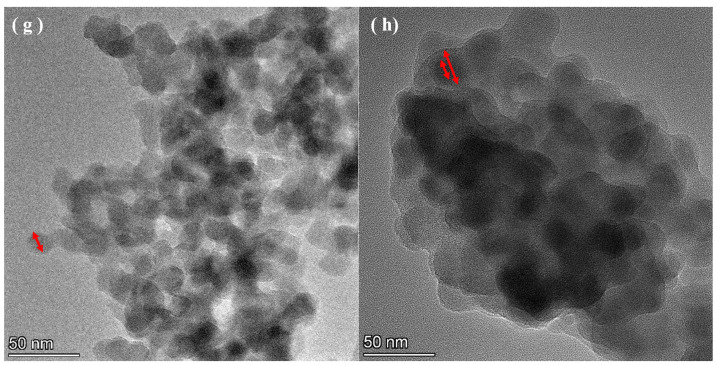
Characterizations of adsorbent: (**a**) FTIR spectra of SiO_2_, SiO_2_–Br and SiO_2_–g–PBMA–b–PDMAEMA. (**b**) TGA curves of SiO_2_, SiO_2_–Br and SiO_2_–g–PBMA–b–PDMAEMA. (**c**) ^1^H NMR spectrum of SiO_2_–g–PBMA–b–PDMAEMA. (**d**) GPC curve of SiO_2_–g–PBMA–b–PDMAEMA. (**e**) XRD patterns of SiO_2_, SiO_2_–g–PBMA–b–PDMAEMA and EBiB–g–PBMA–b–PDMAEMA. (**f**) Nitrogen adsorption–desorption isotherms and the corresponding pore size distribution of SiO_2_–g–PBMA–b–PDMAEMA. (**g**) TEM image of SiO_2_. (**h**) TEM image of SiO_2_–g–PBMA–b–PDMAEMA.

Characterizations of SiO_2_–g–PBMA–b–PDMAEMA are shown in Figure 1. Figure 1a shows the FTIR spectra of SiO_2_, SiO_2_–Br and SiO_2_–g–PBMA–b–PDMAEMA. The peak in SiO_2_ around 3440 cm^−1^ was attributed to SiO–H vibration, and the peaks around 1107 cm^−1^, 800 cm^−1^ and 474 cm^−1^ were attributed to Si–O vibration. Compared with SiO_2_, the new peak in SiO_2_–Br around 2970 cm^−1^ was attributed to C–H vibration in APTES and BiBB, and the new peak of C=O vibration around 1720 cm^−1^ was attributed to the grafting of BiBB. This indicated that the initiator SiO_2_–Br was successfully prepared. SiO_2_–g–PBMA–b–PDMAEMA expressed strong absorption peaks around 2970 cm^−1^ and 1720 cm^−1^, which were attributed to the grafting of PBMA and PDMAEMA, yet the absorption peaks of SiO_2_ around 1107 cm^−1^ and 474 cm^−1^ were still maintained. FTIR results proved the chemical structure of SiO_2_–g–PBMA–b–PDMAEMA, indicating the successful preparation of hybrid adsorbent. Figure 1b shows the TGA curves of SiO_2_, SiO_2_–Br and SiO_2_–g–PBMA–b–PDMAEMA. The weight loss below 120℃ was due to the evaporation of absorbed water. The weight loss from 120 ℃ to 1000 ℃ for SiO_2_ and SiO_2_–Br were 5.14% and 20.35%, respectively, and the graft density of SiO_2_–Br was calculated as 0.73 mmol·g^−1^ by the content of Br. The graft density of SiO_2_–Br laid a foundation for the recipe design of subsequent polymerization. The weight loss from 120 ℃ to 1000 ℃ for SiO_2_–g–PBMA–b–PDMAEMA was 88.69%, which indicated that the grafting percentage of PBMA–b–PDMAEMA was 83.55%. SiO_2_–g–PBMA–b–PDMAEMA had a residual weight of 10.17%, which was attributed to the residual SiO_2_. Figure 1c shows the ^1^H NMR spectrum of SiO_2_–g–PBMA–b–PDMAEMA. The signal of –C–CH_3_ protons was at δ 0.92 ppm (peak a). The signals of –C–CH_2_CH_2_–C– protons in PBMA were at δ 1.39 and 1.61 ppm (peak b). The signal of –CH_2_– protons in the backbone was at δ 1.83 ppm (peak c). The signals of –N–CH_3_ and –N–CH_2_ protons in PDMAEMA were at δ 2.29 and 2.58 ppm (peaks d and e). The signal for –CH_2_ next to –O-C=O was found at δ 4.07 ppm (peak f). ^1^H NMR results further proved the grafting of PBMA–b–PDMAEMA onto SiO_2_, indicating the successful preparation of hybrid adsorbent together with the FTIR results. Figure 1d shows the GPC curve of SiO_2_–g–PBMA–b–PDMAEMA. It represented a number-average molecular weight of 18,541 g·mol^−1^ and a polydispersity index (PDI) of 1.86. This indicated that the measured molecular weight was not far from the theoretical molecular weight of a single arm (27,848 g·mol^−1^) and that the molecular weight distribution was narrow. GPC results verified a typical ATRP process, and the SiO_2_–g–PBMA–b–PDMAEMA was successfully prepared as expected. Figure 1e exhibits XRD patterns of SiO_2_, SiO_2_–g–PBMA–b–PDMAEMA and EBiB–g–PBMA–b–PDMAEMA to confirm the non-crystalline structure of the adsorbent. Pure SiO_2_ showed an amorphous structure with a wide diffraction peak around 22.4° corresponding to the (101) plane [36]. The pure polymer showed an amorphous structure without sharp peaks, which was also reported in the literature [37,38,39]. The hybrid material also showed an amorphous structure, and the effect of polymer grafting onto SiO_2_ could be seen through the peak shift of the (101) plane from 2θ = 22.4° to 2θ = 21.2°. The XRD diffraction peak shifted to a lower angle, indicating an increase in interplanar spacing [40], causing an easier diffusion of adsorbates into the adsorbent’s structure. Figure 1f represents the nitrogen adsorption–desorption isotherms and the corresponding pore size distribution of SiO_2_–g–PBMA–b–PDMAEMA. The hybrid material displayed type-IV N_2_ sorption isotherms with an H3 hysteresis loop, indicating its disordered mesoporous structure [41]. The hybrid material exhibited a lower specific surface area of 79.89 m^2^·g^−1^ but higher adsorption capacity for metal cations than other reported silica-based hybrid materials [41,42], suggesting that the grafted polymer played an important role in adsorption. The hybrid material also exhibited a pore volume of 0.26 cm^3^·g^−1^ and an average mesoporous diameter of 36.38 nm, which was conducive to the diffusion of sodium oleate. Figure 1g,h represent the TEM images of SiO_2_ and SiO_2_–g–PBMA–b–PDMAEMA. Silica exhibited spherical particles with an average diameter of 20 nm. After the grafting of PBMA–b–PDMAEMA chains, the hybrid formed spherical particles with a significantly increased diameter of 30–35 nm. This additionally proved the successful preparation of SiO_2_–g–PBMA–b–PDMAEMA. The aggregation of the hybrid’s particles was reduced compared with pure SiO_2_, and this was because of the grafting of PBMA–b–PDMAEMA chains. The better dispersity in water was favorable to adsorption.

### 3.2. Adsorption Kinetics

**Figure 2:**
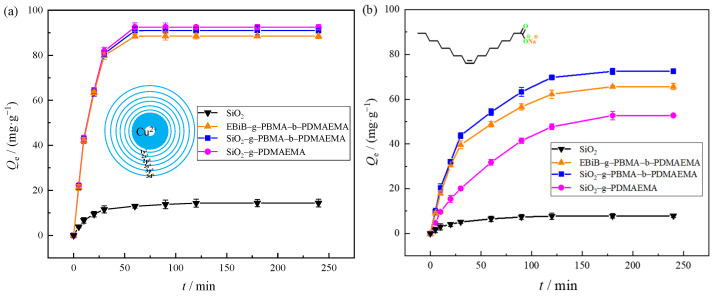
Adsorption kinetics of (**a**) Cu(II) or (**b**) NaOL on SiO_2_, EBiB–g–PBMA–b–PDMAEMA, SiO_2_–g–PBMA–b–PDMAEMA and SiO_2_–g–PDMAEMA (*C*_0_ = 100 mg·L^−1^, pH = 5, *T* = 25 °C, adsorbent concentration = 1 g·L^−1^).

Adsorption kinetics curves are shown in Figure 2. In order to investigate the contribution of SiO_2_ and the grafted polymer to SiO_2_–g–PBMA–b–PDMAEMA adsorption, the absorption capacity of the pure SiO_2_ and pure polymer for both pollutants was taken into account. A detailed recipe for pure polymer (EBiB–g–PBMA–b–PDMAEMA) is listed in Table 2, and the preparation of EBiB–g–PBMA–b–PDMAEMA was simply proved by FTIR (Supporting Information, Figure S2). The adsorption capacity of Cu(II) and sodium oleate on pure silica was small (14.33 mg·g^−1^ for Cu(II) and 7.85 mg·g^−1^ for sodium oleate), while the adsorption capacity of Cu(II) and sodium oleate on pure polymer (88.48 mg·g^−1^ for Cu(II) and 65.67 mg·g^−1^ for sodium oleate) was close to that of SiO_2_–g–PBMA–b–PDMAEMA (91.02 mg·g^−1^ for Cu(II) and 72.47 mg·g^−1^ for sodium oleate). This indicated to us that the grafting of polymer effectively improved the adsorption capacity of the hybrid material.

In particular, to investigate the contribution of hydrophobic PBMA to the multifunctional adsorption of SiO_2_–g–PBMA–b–PDMAEMA, adsorption using SiO_2_–g–PDMAEMA (detailed recipe of polymerization in Table 2) was also discussed. The preparation of SiO_2_–g–PDMAEMA was simply proved by FTIR (Supporting Information, Figure S3). Figure 2a shows the rapid and effective adsorption of Cu(II) by both hybrid adsorbents. The adsorption capacity increased rapidly within 0–30 min and tended to be a constant after 60 min. Therefore, the adsorption equilibrium of Cu(II) on SiO_2_–g–PBMA–b–PDMAEMA was reached within 60 min, providing an experimental parameter for subsequent adsorption isotherms experiments. The equilibrium absorption capacity of Cu(II) on SiO_2_–g–PBMA–b–PDMAEMA was 91.02 mg·g^−1^, which was slightly lower than the adsorption capacity of Cu(II) on SiO_2_–g–PDMAEMA (92.48 mg·g^−1^), indicating a stronger affinity of hydrophilic PDMAEMA to Cu(II). Figure 2b shows that a small quantity of PBMA could effectively improve the adsorption capacity of SiO_2_–g–PBMA–b–PDMAEMA to sodium oleate (NaOL). The equilibrium absorption capacity of NaOL on SiO_2_–g–PBMA–b–PDMAEMA was 72.47 mg·g^−1^, which was much higher than the adsorption capacity of NaOL on SiO_2_–g–PDMAEMA (52.74 mg·g^−1^), indicating a stronger affinity of hydrophobic PBMA to NaOL. The adsorption capacity of NaOL on SiO_2_–g–PBMA–b–PDMAEMA increased rapidly within 0–120 min and tended to be a constant after 180 min. Therefore, the adsorption equilibrium of NaOL on SiO_2_–g–PBMA–b–PDMAEMA was reached within 180 min.

**Figure 3:**
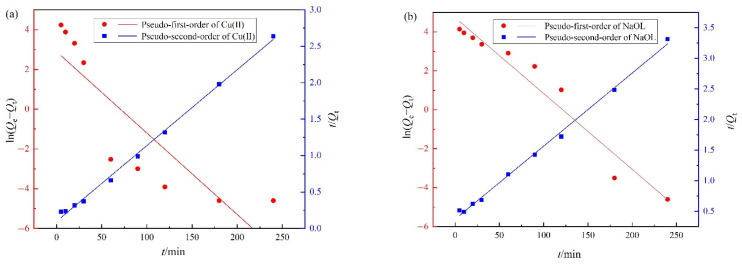
Fitting curves of adsorption kinetics for (**a**) Cu(II) and (**b**) NaOL on SiO_2_–g–PBMA–b–PDMAEMA.

The adsorption kinetics data of Cu(II) and NaOL on SiO_2_–g–PBMA–b–PDMAEMA were fitted to the pseudo-first-order and pseudo-second-order models. The fitting results are shown in Figure 3 and Table 3. The pseudo-second-order model exhibited higher correlation coefficients (*R*^2^ = 0.9966 for Cu(II) and *R*^2^ = 0.9966 for NaOL) and better agreement between experimental adsorption capacity (*Q*_e,exp_ = 91.02 mg·g^−1^ for Cu(II) and *Q*_e,exp_ = 72.47 mg·g^−1^ for NaOL) and calculated adsorption capacity (*Q*_e,cal_ = 96.06 mg·g^−1^ for Cu(II) and *Q*_e,cal_ = 83.75 mg·g^−1^ for NaOL). The adsorption data fitted to the pseudo-second-order model well, indicating that the adsorption rates of Cu(II) and NaOL onto SiO_2_–g–PBMA–b–PDMAEMA were controlled by chemical processes [43].

### 3.3. Adsorption Isotherms

**Figure 4:**
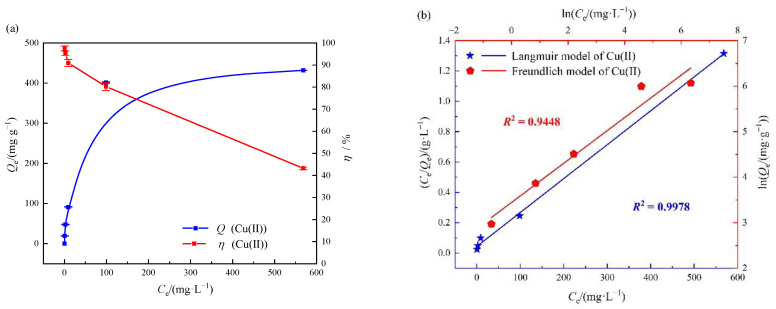
(**a**) Adsorption isotherms and (**b**) Linear fitting plots of Langmuir and Freundlich models of Cu(II) on SiO_2_–g–PBMA–b–PDMAEMA (pH = 5, *T* = 25 ℃, adsorbent concentration = 1 g·L^−1^).

Adsorption isotherm results of Cu(II) on SiO_2_–g–PBMA–b–PDMAEMA are shown in Figure 4 and Table 4. The *Q*_e_ of Cu(II) increased with an increase in equilibrium concentration and then tended to be a constant. The Langmuir adsorption isotherm model exhibited a higher correlation coefficient (*R*^2^ = 0.9978), indicating that Cu(II) was mainly adsorbed by the monolayer. The maximum adsorption capacity of Cu(II) reached 448.43 mg·g^−1^. The Freundlich adsorption isotherm model exhibited an *n* value of 2.15; an *n* value between 2 and 10 indicated that the adsorption of Cu(II) easily occurred at 25 ℃ [44].

**Figure 5:**
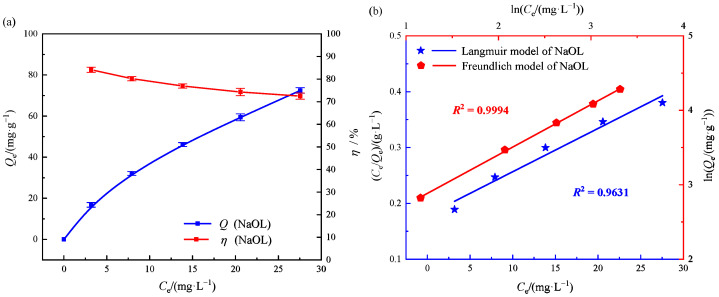
(**a**) Adsorption isotherms and (**b**) Linear fitting plots of Langmuir and Freundlich models of NaOL on SiO_2_–g–PBMA–b–PDMAEMA (pH = 5, *T* = 25 ℃, adsorbent concentration = 1 g·L^−1^).

Adsorption isotherms results of NaOL on SiO_2_–g–PBMA–b–PDMAEMA are shown in Figure 5 and Table 4. Under low initial concentration (*C*_0_ ≤ 100 mg·L^−1^) conditions, the *Q*_e_ of NaOL increased with an increase in equilibrium concentration. The Freundlich adsorption isotherm model exhibited a higher correlation coefficient (*R*^2^ = 0.9994), indicating the multilayered adsorption of NaOL on a heterogeneous surface. The *n* value was 1.48 (*n* > 1), indicating that the adsorption of NaOL on SiO_2_–g–PBMA–b–PDMAEMA was a beneficial adsorption process [45]. The maximum adsorption capacity of NaOL calculated from the Langmuir adsorption isotherm equation reached 129.03 mg·g^−1^.

### 3.4. Adsorption Mechanism

**Figure 6:**
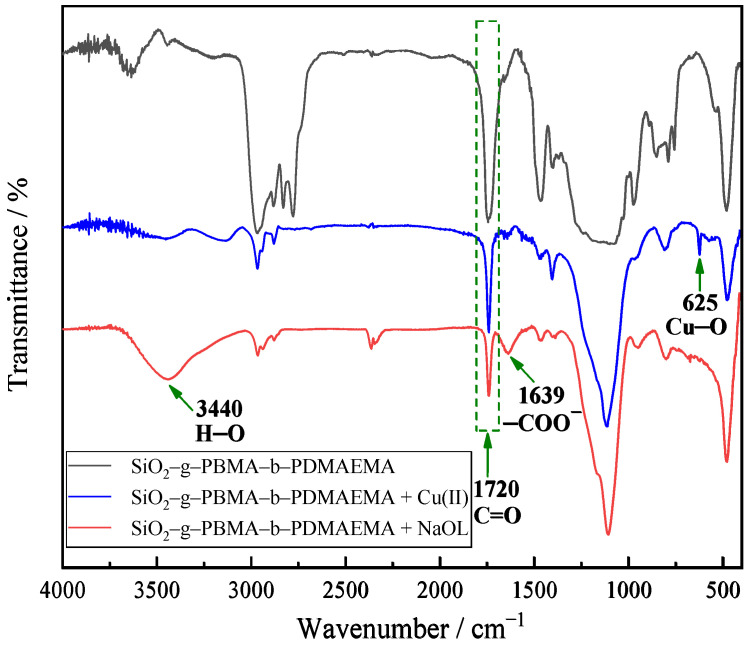
FTIR spectra of complexes of SiO_2_–g–PBMA–b–PDMAEMA with Cu(II) and NaOL.

**Figure 7:**
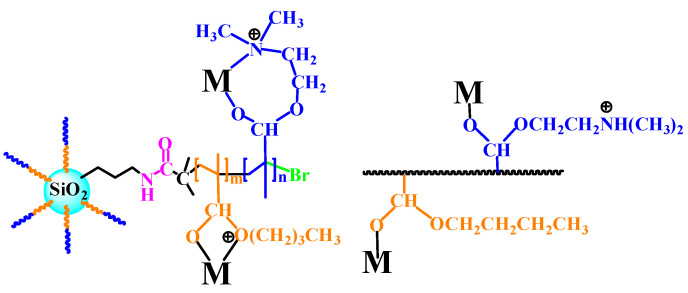
The proposed mechanisms for metal cation (M) adsorption.

**Figure 8:**
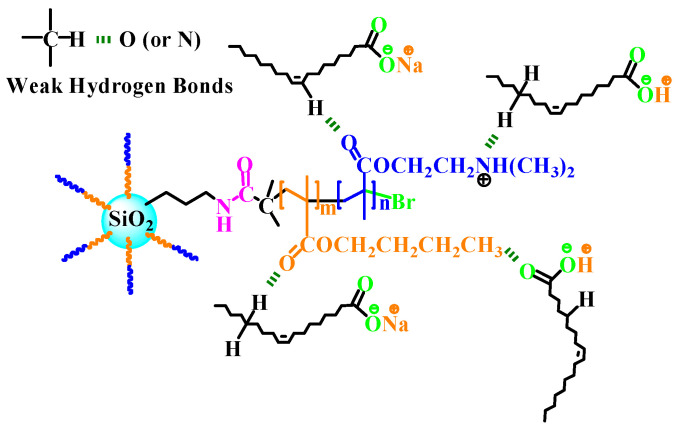
The proposed mechanisms for NaOL adsorption.

FTIR was used to investigate the possible adsorption mechanism (Figure 6). Compared with SiO_2_–g–PBMA–b–PDMAEMA, the peak of C=O vibration around 1720 cm^−1^ in the complex of SiO_2_–g–PBMA–b–PDMAEMA with Cu(II) was significantly weakened, indicating that the oxygen atoms of carbonyl participated in the complexation with Cu(II). The new peak in 625 cm^−1^ was attributed to Cu–O vibration [46]. Combining the results of the pseudo-second-order fitting results (chemical process was the rate-determining step) and Langmuir adsorption isotherm results (monolayer absorption), the proposed mechanisms for Cu(II) adsorption extend to metal cation (M) adsorption, shown in Figure 7. Chelation and complexation were the main driving forces of adsorption. For the FTIR spectrum of SiO_2_–g–PBMA–b–PDMAEMA with NaOL, the peak of C=O vibration around 1720 cm^−1^ was also significantly weakened, indicating that the oxygen atoms of carbonyl participated in the adsorption with NaOL. The new peak of H–O vibration around 3440 cm^−1^ was attributed to the adsorption of oleic acid. This was because at a pH level of 5, a part of sodium oleate was in the form of oleic acid [11,15]. The new peak around 1639 cm^−1^ was attributed to –COO^–^ vibration. Combining the results of the pseudo-second-order fitting results and Freundlich adsorption isotherm results (multilayer adsorption), the proposed mechanisms for NaOL adsorption are shown in Figure 8. The van der Waals force was, of course, a very important driving force for NaOL adsorption. In addition, weak hydrogen bonding [47,48] was a driving force that was easily overlooked. The hydrogen atom attached to the carbon atom had the ability to form weak hydrogen bonds with oxygen and nitrogen atoms, and the weak hydrogen bonds further increased the adsorption capacity of sodium oleate on SiO_2_–g–PBMA–b–PDMAEMA.

### 3.5. Practicability Assessment

**Figure 9:**
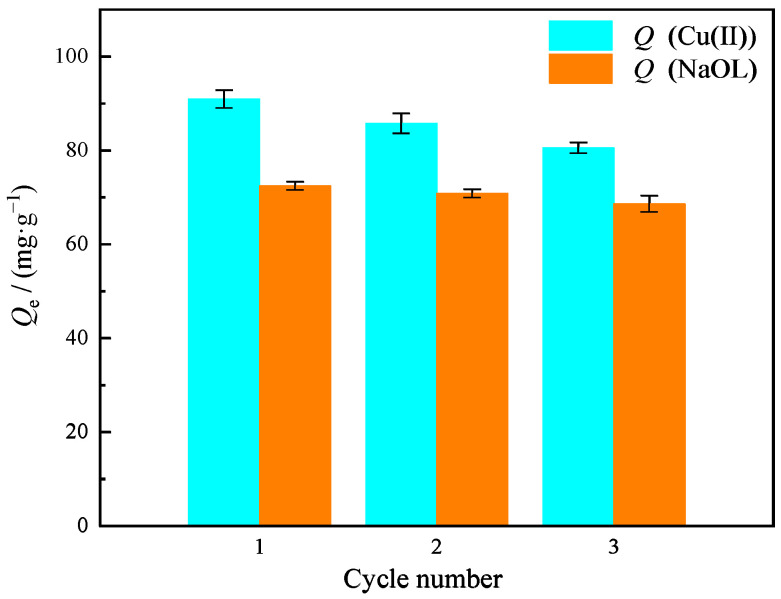
Adsorption–desorption cycles of Cu(II) and NaOL on SiO_2_–g–PBMA–b–PDMAEMA.

Adsorption–desorption cycles (Figure 9) were conducted to evaluate the recyclability of SiO_2_–g–PBMA–b–PDMAEMA. After three cycles, the adsorption capacity of Cu(II) was 80.56 mg·g^−1^, which was 88.58% of the initial adsorption capacity. For NaOL adsorption, the adsorption capacity was 68.59 mg·g^−1^ after three cycles, which was 94.67% of the initial adsorption capacity. These results indicated that the adsorbent was recyclable and of green economic value.

**Figure 10:**
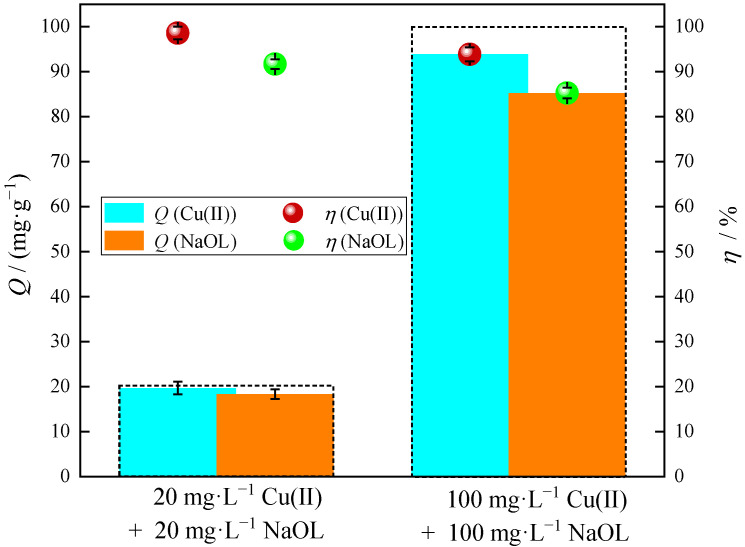
Multicomponent adsorption of Cu(II) and NaOL on SiO_2_–g–PBMA–b–PDMAEMA.

Multicomponent adsorption (Figure 10) was also carried out to investigate the practicability of the adsorbent. The adsorbent showed excellent adsorption capacity for Cu(II) and sodium oleate in the mixed solution. When the initial concentrations of Cu(II) and sodium oleate were both 20 mg·L^−1^, the removal efficiency of Cu(II) reached 98.60% and the removal efficiency of sodium oleate reached 91.70% simultaneously. When the initial concentrations of Cu(II) and sodium oleate were both 100 mg·L^−1^, the removal efficiency of Cu(II) reached 93.88% and the removal efficiency of sodium oleate reached 85.27% simultaneously. The better adsorption of the multicomponent system might be due to the formation of copper oleate, and this did not affect the removal efficiency and practical value of SiO_2_–g–PBMA–b–PDMAEMA.

Generally, the silica-based amphiphilic block copolymer hybrid (SiO_2_–g–PBMA–b–PDMAEMA) showed good performance toward Cu(II) as well as sodium oleate in both single-component and multicomponent adsorption. The adsorption capacity of the hybrid compared to other materials is given in Table 5 [18,19,20,21,23,24,25,29,49,50,51,52,53,54].

## 4. Conclusions

In this work, a silica-based amphiphilic block copolymer, SiO_2_–g–PBMA–b–PDMAEMA, was obtained via SI-ATRP methodology. The hybrid gained excellent adsorption capacity for Cu(II) and sodium oleate, and broadened the application of organic–inorganic hybrids in beneficiation wastewater treatment. All the results can be summarized as follows:

(1) SiO_2_–g–PBMA–b–PDMAEMA was prepared as expected. FTIR and ^1^H NMR directly proved the chemical structure of SiO_2_–g–PBMA–b–PDMAEMA. TGA illustrated that the grafting percentage of PBMA–b–PDMAEMA was 83.55% and the content of SiO_2_ was 10.17%. GPC demonstrated a desired molecular weight of 18,541 g·mol^−1^. TEM also proved the grafting of PBMA–b–PDMAEMA chains. XRD was used to confirm an amorphous structure. Nitrogen adsorption–desorption isotherms exhibited a specific surface area of 79.89 m^2^·g^−1^, a pore volume of 0.26 cm^3^·g^−1^ and an average mesoporous diameter of 36.38 nm;
(2) The effect of hydrophobic chains was investigated. Compared with SiO_2_–g–PDMAEMA, the introduction of a small amount of PBMA did not noticeably affect the adsorption of Cu(II) on SiO_2_–g–PBMA–b–PDMAEMA, but could greatly increase the adsorption of sodium oleate on SiO_2_–g–PBMA–b–PDMAEMA (72.47 mg·g^−1^ with a removal efficiency of 72.47%), which was 1.374 times that of sodium oleate on SiO_2_–g–PDMAEMA (52.74 mg·g^−1^);
(3) Adsorption kinetics showed that the adsorption of Cu(II) and sodium oleate on SiO_2_–g–PBMA–b–PDMAEMA fitted the pseudo-second-order model well, indicating that the adsorption rates were controlled by the chemical process;
(4) Adsorption isotherms of Cu(II) on SiO_2_–g–PBMA–b–PDMAEMA were better described by the Langmuir adsorption isotherm model, indicating that Cu(II) was mainly adsorbed by the monolayer. Adsorption isotherms of sodium oleate on SiO_2_–g–PBMA–b–PDMAEMA were better described by the Freundlich adsorption isotherm model, indicating the multilayered adsorption of sodium oleate on a heterogeneous surface. The maximum adsorption capacity of Cu(II) and sodium oleate calculated by the Langmuir adsorption isotherm equation reached 448.43 mg·g^−1^ and 129.03 mg·g^−1^, respectively;
(5) The adsorption mechanism was investigated. Combining FTIR, adsorption kinetics and adsorption isotherm analysis, chelation and complexation were considered as the main driving forces of Cu(II) adsorption on SiO_2_–g–PBMA–b–PDMAEMA. The van der Waals force as well as weak hydrogen bonding were considered as the main driving forces of sodium oleate adsorption on SiO_2_–g–PBMA–b–PDMAEMA;
(6) The practical value of the adsorbent was evaluated. SiO_2_–g–PBMA–b–PDMAEMA was recyclable with a 88.58% adsorption capacity for Cu(II) and a 94.67% adsorption capacity for sodium oleate after three cycles, and the adsorbent showed excellent multicomponent adsorption for Cu(II) and sodium oleate in the mixed solution.

Further research will be conducted on simultaneous adsorption of various pollutants.

## Figures and Tables

**Table 1:** Materials and the corresponding purification methods.

Materials	Abbreviations	Purity	Purification Methods	Suppliers
Nano-silica	SiO_2_	>99%wt, a mean particle diameter of 20 nm and a specific surface area of 120 m^2^·g^−1^		Hai Tai Nano (Nanjing, China)
(3-Aminopropyl) triethoxysilane	APTES	99%		Aladdin (Shanghai, China)
2-Bromoisobutyrylbromide	BiBB	98%		Aladdin
Sodium hydroxide	NaOH	97%		Aladdin
Calcium hydride	CaH_2_	95%		Aladdin
Triethylamine	TEA	99%	dried over CaH_2_ and distilled under reduced pressure	Aladdin
Butyl methacrylate	BMA	99%	rinsed with 5 wt % NaOH and dried over CaH_2_	Aladdin
2-(Dimethylamino)ethylmethacrylate	DMAEMA	99%	dried over CaH_2_ and distilled under reduced pressure	Aladdin
Tetrahydrofuran	THF	99%	dried over CaH_2_ and distilled under reduced pressure	Aladdin
Cyclohexanone	CYC	99.5%	dried over CaH_2_ and distilled under reduced pressure	Aladdin
Cuprous chloride	CuCl	97%	[32]	Aladdin
Copper chloride	CuCl_2_	98%		Aladdin
N,N,N′,N′,N″-Pentamethyldiethylenetriamine	PMDETA	99%		Aladdin
Ethyl α-bromoisobutyrate	EBiB	98%		Aladdin
Hydrofluoric acid	HF	40%		Aladdin
Ethanol		99.5%		Aladdin
Sodium oleate	NaOL	97%		Aladdin
Copper(II) sulfate pentahydrate	CuSO_4_·5H_2_O,	99%		Chengdu Kelong Chemical Co., Ltd. (Chengdu, China)
Nitric acid	HNO_3_	68%		Foshan Huaxisheng Chemical Co., Ltd. (Foshan, China)

**Table 2:** Detailed recipes of polymerization for prepared samples.

Sample	SiO_2_–Br /mmol	EBiB /mmol	BMA /mmol	DMAEMA /mmol	CuCl /mmol	CuCl_2_ /mmol	PMDETA /mmol	Cyclohexanone /g
SiO_2_–g–PBMA–b–PDMAEMA	0.73		21.90	109.50	0.73	0.073	0.73	32.31
SiO_2_–g–PDMAEMA	0.73			131.40	0.73	0.073	0.73	32.80
EBiB–g–PBMA–b–PDMAEMA		0.73	21.90	109.50	0.73	0.073	0.73	31.00

Note: dosage of cyclohexanone was calculated based on a solid content of 40%.

**Table 3:** Fitting parameters of adsorption kinetics for Cu(II) and NaOL on SiO_2_–g–PBMA–b–PDMAEMA.

Systems	*Q*_e,exp_ /mg·g^−1^	Pseudo-First-Order			Pseudo-Second-Order		
		*Q*_e,cal_ /mg·g^−1^	*k*_1_ /min^−1^	*R* ^2^	*Q*_e,cal_ /mg·g^−1^	*k*_2_ /g·mg^−1^·min^−1^	*R* ^2^
Cu(II)	91.02	18.10	0.0410	0.7236	96.06	0.0011	0.9966
NaOL	72.47	113.58	0.0390	0.9456	83.75	0.0004	0.9966

**Table 4:** Adsorption isotherm parameters for the adsorption of Cu(II) and NaOL on SiO_2_–g–PBMA–b–PDMAEMA.

Systems	Langmuir			Freundlich		
	*Q*_m_ /mg·g^−1^	*K*_L_ /L·mg^−1^	*R* ^2^	*n*	*K*_f_	*R* ^2^
Cu(II)	448.43	0.0509	0.9978	2.15	31.3643	0.9448
NaOL	129.03	0.0433	0.9631	1.48	7.8096	0.9994

**Table 5:** Comparison of Adsorption Capacity.

Adsorbent Material	Experimental Conditions	Cu(II)/ mg·g^−1^	Sodium Oleate/ mg·g^−1^	References
activated carbon	pH = 5, *T* = 27 °C, *C*_0_ = 2 mg L^−1^, *C*_a_ = 1 g·L^−1^	1.581		[18]
carbon nanocomposites	pH = 6.8, *T* = 20 °C	256 *		[19]
amino-Fe(III)-functionalized mesoporous silica	pH = 5.5, *T* = 25 °C, *C*_a_ = 1 g·L^−1^	475.1 *		[20]
natural zeolite	pH = 4.5, *T* = 22 °C, *C*_0_ = 20 mg L^−1^, *C*_a_ = 37 g·L^−1^	3.37		[21]
halloysite nanotube–alginate hybrid beads	*T* = room temperature, *C*_0_ = 100 mg L^−1^	74.13		[23]
PDMAEMA/SiO_2_	pH = 5.0, *T* = 30 °C, *C*_a_ = 4 g·L^−1^	20		[29]
chitosan using tri-sodium citrate and epichlorohydrin as cross-linkers	pH = 6.0, *T* = 25 °C, *C*_a_ = 2 g·L^−1^	151.52 *		[49]
functionalized maghemite nanoparticles	pH = 5.5, *T* = 25 °C, *C*_0_ = 40 mg L^−1^, *C*_a_ = 0.56 g·L^−1^	88.2		[50]
succinylated mercerized cellulose modified with triethylenetetramine	pH = 5.5, *C*_a_ = 1 g·L^−1^	69.4 *		[51]
diethylenetriamine-bacterial cellulose	pH = 4.5, *T* = 25 °C, *C*_a_ = 1 g·L^−1^	63.09 *		[52]
mesoporous nanocellulose/sodium alginate/carboxymethyl–chitosan gel beads	pH = 5, *T* = 30 °C, *C*_a_ = 0.6 g·L^−1^	169.94 *		[53]
beta-cyclodextrin polymers	pH = 5.0, *T* = 25 °C, *C*_0_ = 200 mg L^−1^, *C*_a_ = 0.5 g·L^−1^	164.43		[54]
modified Ca-montmorillonite	pH = 5, *T* = 25 °C, *C*_0_ = 100 mg L^−1^, *C*_a_ = 1.5 g·L^−1^		69.199 (22. 73 mmol/100 g)	[24]
Zr-Modified Phosphogypsum/Fly Ash Composite	pH = 7.19, *T* = 25 °C, *C*_a_ = 5 g·L^−1^		14. 376 *	[25]
SiO_2_–g–PBMA–b–PDMAEMA	pH = 5, *T* = 25 °C, *C*_a_ = 1 g·L^−1^	448.43 *	129.03 *	This study

Note: *C*_0_ refers to the initial concentration of Cu(II) or sodium oleate. *C*_a_ refers to the concentration of adsorbent. * refers to the maximum adsorption capacity calculated from the Langmuir adsorption isotherm equation.

## Data Availability

Data presented in this study are available on request from the first author.

## Supplementary Materials

The following supporting information can be downloaded at: https://www.mdpi.com/article/10.3390/polym14194187/s1, Figure S1: Standard curve of sodium oleate. Figure S2: FTIR spectrum of EBiB–g–PBMA–b–PDMAEMA. Figure S3: FTIR spectrum of SiO_2_–g–PDMAEMA.

## References

[1] Florea R.M., Stoica A.I., Baiulescu G.E., Capotă P. (2005). Water pollution in gold mining industry: A case study in Roia Montan district, Romania. Environ. Geol..

[2] Jabłońska-Czapla M., Nocoń K., Szopa S., Łyko A. (2016). Impact of the Pb and Zn ore mining industry on the pollution of the Biała Przemsza River, Poland. Environ. Monit. Assess..

[3] Ali I., Gupta V.K. (2006). Advances in water treatment by adsorption technology. Nat. Protoc..

[4] Demirbas A. (2008). Heavy metal adsorption onto agro-based waste materials: A review. J. Hazard. Mater..

[5] Benavente M., Moreno L., Martinez J. (2011). Sorption of heavy metals from gold mining wastewater using chitosan. J. Taiwan. Inst. Chem. E.

[6] Nguyen H.T.H., Nguyen B., Duong T.T., Bui A.T.K., Nguyen H.T.A., Cao T.H., Mai N.T., Nguyen K.M., Pham T.T., Kim K.-W. (2019). Pilot-scale removal of arsenic and heavy metals from mining wastewater using adsorption combined with constructed wetland. Minerals.

[7] Ören A.H., Kaya A. (2006). Factors affecting adsorption characteristics of Zn^2+^ on two natural zeolites. J. Hazard. Mater..

[8] Rolland N., Larocque I. (2007). The efficiency of kerosene flotation for extraction of chironomid head capsules from lake sediments samples. J. Paleolimnol..

[9] Tan Y., Chen T., Zheng S., Sun Z., Li C. (2021). Adsorptive and photocatalytic behaviour of PANI/TiO_2_/metakaolin composites for the removal of xanthate from aqueous solution. Miner. Eng..

[10] Lin W., Tian J., Ren J., Xu P., Dai Y., Sun S., Wu C. (2016). Oxidation of aniline aerofloat in flotation wastewater by sodium hypochlorite solution. Environ. Sci. Pollut. R..

[11] Xue J., Zhong H., Wang S. (2019). Removal of sodium oleate from synthetic manganese leaching solution by coagulation-dissolved air flotation. J. Environ. Manag..

[12] Ashkenazi D., Taxel I., Tal O. (2015). Archeometallurgical characterization of Late Roman- and Byzantine-period Samaritan magical objects and jewelry made of copper alloys. Mater. Charact..

[13] Unger N., Gough O. (2007). Life cycle considerations about optic fibre cable and copper cable systems: A case study. J. Clean. Prod..

[14] Zhou Y.Y., Zhang F.F., Tang L., Zhang J.C., Zeng G.M., Luo L., Liu Y.Y., Wang P., Peng B., Liu X.C. (2017). Simultaneous removal of atrazine and copper using polyacrylic acid-functionalized magnetic ordered mesoporous carbon from water: Adsorption mechanism. Sci. Rep..

[15] Li Z., Rao F., García R.E., Li H., Song S. (2018). Partial replacement of sodium oleate using alcohols with different chain structures in malachite flotation. Miner. Eng..

[16] Makuei F., Tadesse B., Albijanic B., Browner R. (2018). Electroflotation of ultrafine chalcopyrite particles with sodium oleate collector. Miner. Eng..

[17] Sindu P.A., Gautam P. (2017). Studies on the biofilm produced by Pseudomonas aeruginosa grown in different metal fatty acid salt media and its application in biodegradation of fatty acids and bioremediation of heavy metal ions. Can. J. Microbiol..

[18] Onundi Y.B., Mamun A.A., Khatib M.F.A., Ahmed Y.M. (2010). Adsorption of copper, nickel and lead ions from synthetic semiconductor industrial wastewater by palm shell activated carbon. Int. J. Environ. Sci. Tech..

[19] Dichiara A.B., Webber M.R., Gorman W.R., Rogers R.E. (2015). Removal of Copper Ions from Aqueous Solutions via Adsorption on Carbon Nanocomposites. ACS Appl. Mater. Interfaces..

[20] Zhang Z., Liu H., Wu L., Lan H., Qu J. (2015). Preparation of amino-Fe(III) functionalized mesoporous silica for synergistic adsorption of tetracycline and copper. Chemosphere.

[21] Motsi T., Rowson N.A., Simmons M.J.H. (2009). Adsorption of heavy metals from acid mine drainage by natural zeolite. Int. J. Miner. Process..

[22] Qureshi F., Memon S.Q., Khuhawar M.Y., Jahangir T.M. (2021). Removal of Co^2+^, Cu^2+^ and Au^3+^ ions from contaminated wastewater by using new fluorescent and antibacterial polymer as sorbent. Polym. Bull..

[23] Wang Y., Zhang X., Wang Q., Zhang B., Liu J. (2014). Continuous fixed bed adsorption of Cu(II) by halloysite nanotube-alginate hybrid beads: An experimental and modelling study. Water. Sci. Technol..

[24] Ren R., Zhang Q., Shi Q., Li C., Wang X., Meng Y. (2015). Sodium oleate adsorption by modified Ca-montmorillonite under acid condition. Chin. J. Environ. Eng..

[25] Jia Z., Liu Z., Song Y., Fan X. (2020). Adsorption of sodium oleate in mineral processing wastewater by zr modified phosphogypsum/fly ash composite. Mater. Rep..

[26] Moon J.K., Kim K.W., Jung C.H., Shul Y.G., Lee E.H. (2000). Preparation of Organic-Inorganic Composite Adsorbent Beads for Removal of Radionuclides and Heavy Metal Ions. J. Radioanal. Nucl. Ch..

[27] Meyer T., Prause S., Spange S., Friedrich M. (2001). Selective ion pair adsorption of cobalt and copper salts on cationically produced poly(1,3-divinylimidazolid-2-one)/silica hybrid particles. J. Colloid. Interface. Sci..

[28] Samiey B., Cheng C.H., Wu J. (2014). Organic-inorganic hybrid polymers as adsorbents for removal of heavy metal ions from solutions: A review. Materials.

[29] Gao B., Chen Y., Zhang Z. (2010). Preparation of functional composite grafted particles PDMAEMA/SiO_2_ and preliminarily study on functionality. Appl. Surf. Sci..

[30] Zhou W., Liu H., Ye H., Cui H., Wang R., Li J., Zhang X. (2013). Synthesis and adsorption behaviors of poly(2-(dimethylamino)ethyl methacrylate) brushes on silica particles by surface-initiated atom transfer radical polymerization. Powder. Technol..

[31] Wang J., Zheng Y., Kang Y., Wang A. (2013). Investigation of oil sorption capability of PBMA/SiO_2_ coated kapok fiber. Chem. Eng. J..

[32] Pan A.Z., He L. (2014). Fabrication pentablock copolymer/silica hybrids as self-assembly coatings. J. Colloid Interface Sci..

[33] Qu J., Yang Q., Gong W., Li M., Cao B. (2022). Simultaneous removal of Cr(VI) and phenol from water using silica-di-block polymer hybrids: Adsorption kinetics and thermodynamics. Polymers.

[34] Konggidinata M.I., Chao B., Lian Q., Subramaniam R., Zappi M., Gang D.D. (2017). Equilibrium, kinetic and thermodynamic studies for adsorption of BTEX onto Ordered Mesoporous Carbon (OMC). J. Hazard. Mater..

[35] Ezzeddine Z., Batonneau-Gener I., Pouilloux Y., Hamad H., Saad Z. (2021). The applicability of as synthesized mesoporous carbon CMK-3 as a heavy metals adsorbent: Application to real water samples. Energ. Source. Part. A.

[36] Piroonpan T., Huajaikaew E., Katemake P., Pasanphan W. (2020). Surface modification of SiO_2_ nanoparticles with PDMAEMA brushes and Ag nanoparticles as antifungal coatings using electron beam assisted synthesis. Mater. Chem. Phys..

[37] Arunkumar R., Babu R.S., Rani M.U. (2017). Investigation on Al_2_O_3_ doped PVC–PBMA blend polymer electrolytes. J. Mater. Sci. Mater. Electron..

[38] Suhailath K., Ramesan M.T. (2020). Theoretical and experimental studies on DC conductivity and temperaturedependent AC conductivity of poly(butyl methacrylate)/Nd-TiO_2_ nanocomposites. J. Thermoplast. Compos..

[39] Kumar K., Mogha N.K., Yadav R., Venkatesu P. (2020). Insulin-induced conformational transition of fluorescent copolymer: A perspective of self-assembly between protein and micellar solution of smart copolymer. Phys. Chem. Chem. Phys..

[40] Liu Y., Huo R., Qin H., Li X., Wei D., Zeng T. (2020). Overcharge investigation of degradations and behaviors of large format lithium ion battery with Li(Ni_0.6_Co_0.2_Mn_0.2_)O_2_ cathode. J. Energy. Storage..

[41] Pavan F.A., Costa T.M.H., Benvenutti E.V. (2003). Adsorption of CoCl_2_, ZnCl_2_ and CdCl_2_ on aniline/silica hybrid material obtained by sol–gel method. Colloids Surface A.

[42] Liang X., Xu Y., Sun G., Wang L., Sun Y., Qin X. (2009). Preparation, characterization of thiol-functionalized silica and application for sorption of Pb^2+^ and Cd^2+^. Colloids Surface A.

[43] Zeng G.M., Liu Y.Y., Tang L., Yang G.D., Pang Y., Zhang Y., Zhou Y.Y., Li Z., Li M.Y., Lai M.Y. (2015). Enhancement of Cd(II) adsorption by polyacrylic acid modified magnetic mesoporous carbon. Chem. Eng. J..

[44] Yang F.J., Ma C.H., Yang L., Zhao C.J., Zhang Y., Zu Y.G. (2012). Enrichment and purification of deoxyschizandrin and γ-schizandrin from the extract of schisandra chinensis fruit by macroporous resins. Molecules.

[45] Malakootian M., Mansoorian H.J., Yari A.R. (2014). Removal of reactive dyes from aqueous solutions by a non-conventional and low cost agricultural waste: Adsorption on ash of Aloe Vera plant. Iran. J. Health Saf. Environ..

[46] O’Keeffe M. (1963). Infrared optical properties of cuprous oxide. J. Chem. Phys..

[47] Desiraju G.R. (2005). C–H⋯O and other weak hydrogen bonds. From crystal engineering to virtual screening. Chem. Commun..

[48] Takahashi O., Kohno Y., Nishio M. (2010). Relevance of weak hydrogen bonds in the conformation of organic compounds and bioconjugates: Evidence from recent experimental data and high-level ab initio MO calculations. Chem. Rev..

[49] Sun Y.F., Liu Y., Yang Y., Li J.J. (2014). Enhancing adsorption of Pb (II) and Cu (II) ions onto chitosan using tri-sodium citrate and epichlorohydrin as cross-linkers. Desalin. Water. Treat..

[50] Guivar J.A.R., Sadrollahi E., Menzel D., Fernandes E., López E.O., Torres M.A.M., Arsuaga J.M., Arencibia A., Litterst F.J. (2017). Magnetic, structural and surface properties of functionalized maghemite nanoparticles for copper and lead adsorption. RSC Adv..

[51] Gurgel L.V.A., Gil L.F. (2009). Adsorption of Cu(II), Cd(II), and Pb(II) from aqueous single metal solutions by succinylated mercerized cellulose modified with triethylenetetramine. Carbohydr. Polym..

[52] Shen W., Chen S., Shi S., Li X., Zhang X., Hu W., Wang H. (2009). Adsorption of Cu(II) and Pb(II) onto diethylenetriamine-bacterial cellulose. Carbohydr. Polym..

[53] Li W., Zhang L., Hu D., Yang R., Zhang J., Guan Y., Lv F., Gao H. (2021). A mesoporous nanocellulose/sodium alginate/carboxymethyl-chitosan gel beads for efficient adsorption of Cu^2+^ and Pb^2+^. Int. J. Biol. Macromol..

[54] He J., Li Y., Wang C., Zhang K., Lin D., Kong L., Liu J. (2017). Rapid adsorption of Pb, Cu and Cd from aqueous solutions by beta-cyclodextrin polymers. Appl. Surf. Sci..

