# Supplementary material for: Multifunctional Silica-Based Amphiphilic Block Copolymer Hybrid for Cu(II) and Sodium Oleate Adsorption in Beneficiation Wastewater
Source: Polymers (Basel). 2022 Oct 6;14(19):4187. doi: 10.3390/polym14194187 (PMC9571196; doi:10.3390/polym14194187)
Supplement: Supplementary file 1 [file polymers-14-04187-s001.zip › polymers-1919717-SM.pdf]

Supporting Information

Multifunctional Silica-Based Amphiphilic Block Copolymer Hybrid for Cu(Ⅱ) and Sodium Oleate Adsorption in Beneficiation Wastewater

Jia Qu * , Liangliang Chang, Mingbao Liu, Baoyue Cao, Meilan Li,

Qiang Yang and Wei Gong

Shaanxi Key Laboratory of Comprehensive Utilization of Tailings Resources, Shaanxi Engineering Research Center for Mineral Resources Clean & Efficient Conversion and New Materials, Shangluo University, Shangluo 726000; 231026@slxy.edu.cn (L.C.); lmb74@163.com (M.L.); cby0406@163.com (B.C.); liecho2009@163.com (M.L.); yq_sust@163.com (Q.Y.); gongwei-com@escience.cn (W.G.)

* Correspondence: 231034@slxy.edu.cn; Tel.: +86-0914-2986027

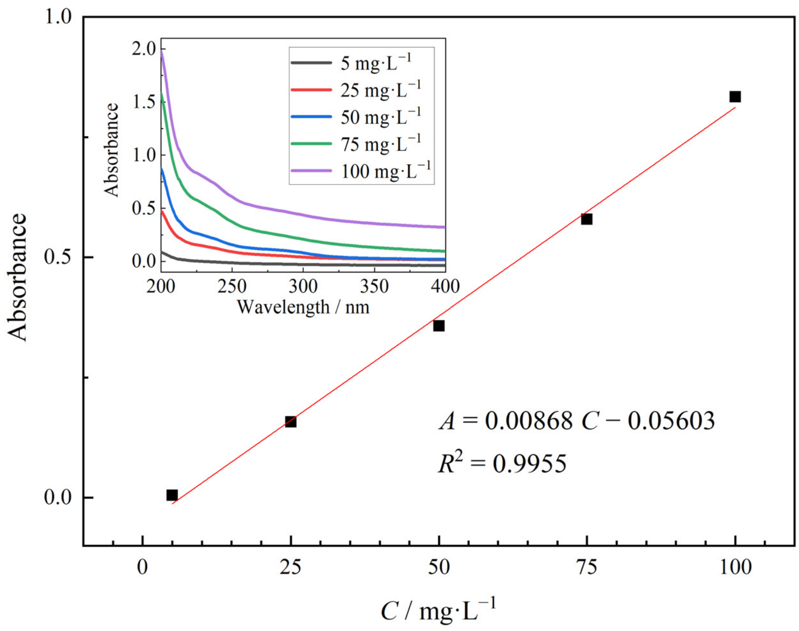

Figure S1. Standard curve of sodium oleate.

Sodium oleate solutions of 5, 25, 50, 75 and 100 mg·L^{-1} were prepared with deionized water. The absorbance of sodium oleate was determined using an ultraviolet-visible spectrophotometer (UV-Vis, Cary 5000, Agilent, Palo Alto, USA). The absorption curves of sodium oleate solutions (inset picture) showed a maximum absorption wavelength of 225 nm, which was consistent with the existing literatures [1,2]. The relation between the concentration of sodium oleate and the absorbance was found to be linear in the range of 5.0 – 100.0 mg·L^{-1}. Since the correlation coefficient was 0.9955, the equation (A = 0.00868 C − 0.05603) could be used as the standard curve equation for the determination of sodium oleate concentration.

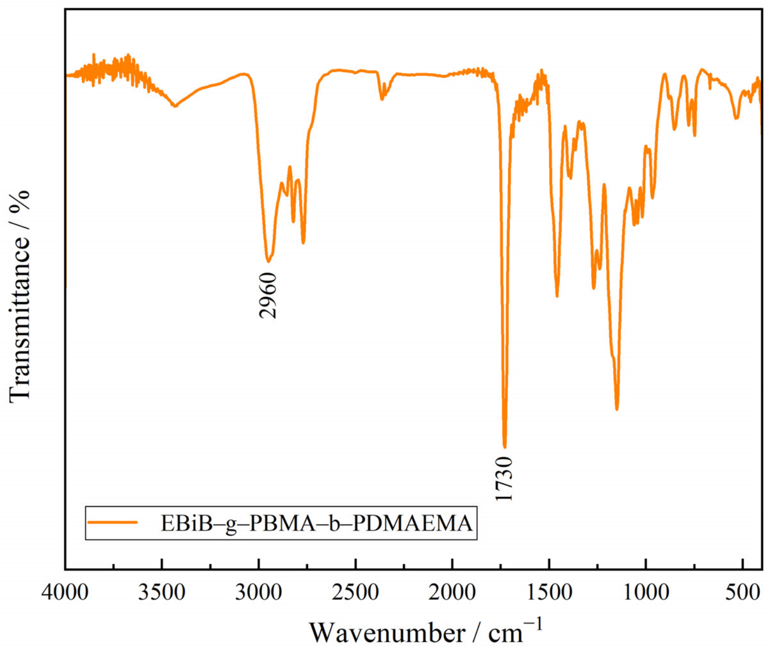

Figure S2. FTIR spectrum of EBiB–g–PBMA–b–PDMAEMA.

The preparation of EBiB–g–PBMA–b–PDMAEMA was simply observed by FTIR. The peak around 2960 cm^{-1} was attributed to the C-H vibration, and the peak around 1730 cm^{-1} was attributed to the C=O vibration. In addition, C=C vibration around 1650 cm^{-1} did not appear in the spectrum, indicating that the polymerization was successful.

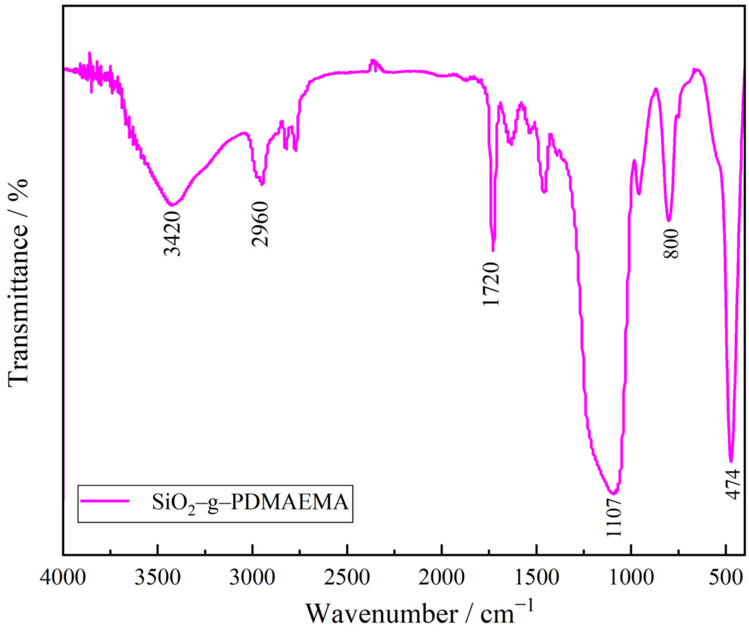

Figure S3. FTIR spectrum of SiO_2–g–PDMAEMA.

The preparation of SiO_2–g–PDMAEMA was simply proved by FTIR. The peak around 3420 cm^{-1} was mainly attributed to the water absorbed physically because of the hydrophilicity of PDMAEMA as well as SiO_2. The peaks around 1107 cm^{-1}, 800 cm^{-1} and 474 cm^{-1} were attributed to the Si-O vibration. Compared with SiO_2, the new peak in SiO_2–g–PDMAEMA around 2960 cm^{-1} was attributed to the C-H vibration, and the new peak around 1720 cm^{-1} was attributed to the C=O vibration. The introduction of C-H and C=O indicated that PDMAEMA was successfully grafted onto the surface of SiO_2.

References

1. Ren, R.; Zhang, Q.; Shi, Q.; Li, C.; Wang, X.; Meng, Y. Sodium oleate adsorption by modified Ca-montmorillonite under acid condition. *Chinese Journal of Environmental Engineering*, **2015**, *9(9)*, 4273–4280. (in Chinese)

2. Jia, Z.; Liu, Z.; Song, Y.; Fan X. Adsorption of sodium oleate in mineral processing wastewater by zr modified phosphogypsum /fly ash composite. *Materials reports*, **2020**, *34(4)*, 07015–07019. (in Chinese)
